# Virus-Specific Stem Cell Memory CD8+ T Cells May Indicate a Long-Term Protection against Evolving SARS-CoV-2

**DOI:** 10.3390/diagnostics13071280

**Published:** 2023-03-28

**Authors:** Milena Aleksova, Yana Todorova, Radoslava Emilova, Magdalena Baymakova, Nina Yancheva, Radina Andonova, Anelia Zasheva, Alba Grifoni, Daniela Weiskopf, Alessandro Sette, Maria Nikolova

**Affiliations:** 1 Immunology Department, National Center of Infectious and Parasitic Diseases, 1000 Sofia, Bulgaria; 2 Department of Infectious Diseases, Military Medical Academy, 1000 Sofia, Bulgaria; 3 Specialized Hospital for Active Treatment of Infectious and Parasitic Diseases, 1000 Sofia, Bulgaria; 4 Center for Infectious Disease and Vaccine Research, La Jolla Institute for Immunology (LJI), Conl Public Health, University of California San Diego (UCSD), San Diego, CA 92037, USA; 5 Department of Pathology, University of California La Jolla, San Diego, CA 92093, USA

**Keywords:** SARS-CoV-2, B.1.1.7 (alpha) variant, B.1.617.2 (delta) variant, biomarkers, stem cell-like memory T cells (T_SCM_)

## Abstract

Immune memory to SARS-CoV-2 is key for establishing herd immunity and limiting the spread of the virus. The duration and qualities of T-cell-mediated protection in the settings of constantly evolving pathogens remain an open question. We conducted a cross-sectional study of SARS-CoV-2-specific CD4+ and CD8+ T-cell responses at several time points over 18 months (30–750 days) post mild/moderate infection with the aim to identify suitable methods and biomarkers for evaluation of long-term T-cell memory in peripheral blood. Included were 107 samples from 95 donors infected during the periods 03/2020–07/2021 and 09/2021–03/2022, coinciding with the prevalence of B.1.1.7 (alpha) and B.1.617.2 (delta) variants in Bulgaria. SARS-CoV-2-specific IFNγ+ T cells were measured in ELISpot in parallel with flow cytometry detection of AIM+ total and stem cell-like memory (T_SCM_) CD4+ and CD8+ T cells after in vitro stimulation with peptide pools corresponding to the original and delta variants. We show that, unlike IFNγ+ T cells, AIM+ virus-specific CD4+ and CD8+ T_SCM_ are more adequate markers of T cell memory, even beyond 18 months post-infection. In the settings of circulating and evolving viruses, CD8+ T_SCM_ is remarkably stable, back-differentiated into effectors, and delivers immediate protection, regardless of the initial priming strain.

## 1. Introduction

The T-cell response is decisive for the issue of acute viral infection. Virus-specific antibodies are efficient in a narrow time window, against free viral particles in blood or on mucosal surfaces. After viral entry, CD4+ and CD8+ virus-specific T cell effectors can eliminate infected cells through direct or IFNγ—mediated cytotoxicity, preventing viral spread and development of complications. CD4+ T cells induce and maintain the affinity maturation of virus-specific antibodies. Virus-induced T cells with inhibitory potential back-regulate immune inflammation. Most importantly, a protective immune response comprises the generation of differentiated long-living memory T cells that react quickly and efficiently in case of reinfection. The formation of efficient and lasting T cell memory after natural infection or vaccination is important for long-term individual and community protection [1,2]

After SARS-CoV-2 emerged, the nature of SARS-CoV-2-specific responses became a matter of legitimate interest. Adaptive immunity to SARS-CoV-2 relies on virus-neutralizing antibodies (Nabs), and virus-specific CD4+ and CD8+ T cells and a number of longitudinal studies were launched to assess the dynamics, longevity, and quality of these responses depending on the type of exposure. It is now known that most patients with confirmed SARS-CoV-2 infection seroconvert, and after an initial decline, Nabs titers are relatively stable for at least 12 months [3,4,5,6]. However, antibody responses are preceded and accompanied by polyfunctional, mainly interferon-γ -secreting CD8+ and CD4+ T cell responses, stable during convalescence, the latter predicting the antibody neutralization breadth and maintenance [7,8,9,10].

It was soon clear that alongside effector responses, immune memory to SARS-CoV-2 is established both after infection and vaccination. Virus-specific memory B cells, antibodies, and memory T cells were detected in mild COVID-19 cases at 90 days post-infection [11]. Several studies are now available that have assessed T cell and B cell memory at longer terms. Sekine et al. described robust memory T-cell responses 49–64 days after an asymptomatic or mild infection, even in the absence of detectable circulating antibodies specific for SARS-CoV-2 [12]. At 6 months post-infection, SARS-CoV-2-specific memory CD4+ and CD8+ T cell responses were established in 90% and 70% of tested subjects, respectively [13,14], and their half-lives were estimated to be about 3–5 months [14]. Finally, Jung et al. demonstrated that SARS-CoV-2-specific CD4+ and CD8+ T cell responses in COVID-19 convalescent patients are maintained up to 317 days post-symptom onset (DPSO), regardless of the severity of COVID-19 [15]. Although these studies propose that SARS-CoV-2-specific memory would resemble that of SARS rather than the quasi-non-existent memory induced by non-SARS-CoVs, a number of issues await elucidation [9].

COVID-19 immune memory is characterized by heterogeneity in ranges and dynamics [9], determined by a number of factors. The unprecedented measures against the pandemic, including quickly developed and massively applied vaccines, and targeted treatment, had an immense impact both on collective immunity and on SARS-CoV-2 evolution. The nature and times of exposure (infection, vaccination, or both), the viral load/severity and treatment of infection, the infecting/re-infecting variant(s), and the particular epidemiological settings are the most important factors that determine the qualities of protection, and the subsets-carriers of long-term memory [12,16,17].

Depending on the efficiency of the initial immune response and viral clearance, virus-specific effector T cells evolve into memory subsets with differing phenotypic and functional characteristics, as demonstrated for flu, EBV, CMV or HBV [18,19]. Thus, assessment of SARS-CoV-2-specific immune memory may require different approaches as compared to early virus-specific responses.

Among the memory subsets, stem cell-like memory T cells (T_SCM_) are particularly attractive, as they possess self-renewal capacity and multipotency to repopulate the broad spectrum of memory and effector T cell subsets with different specificity, required for long-term protection [20]. For example, memory T cells following vaccination with live attenuated yellow fever virus (YFV) exhibit stem cell-like properties and mediate lifelong protection [21]. Few published studies have reported on the differentiation of SARS-CoV-2-specific T_SCM_ following recovery from COVID-19 [12,15,19], and no one has looked at their long-term evolution and variant-specific responses. 

In the present study, we extend the knowledge about SARS-CoV-2 specific T cell response over 18 months post-infection in evolving epidemiological settings and variant dominance. We aimed to identify suitable methods and biomarkers for the long-term evaluation of T-cell memory in peripheral blood, with particular attention to T_SCM_ cells.

## 2. Materials and Methods

### 2.1. Patients and Samples

A total of 95 convalescent patients with PCR-confirmed SARS-CoV-2 infection that have been diagnosed with mild to moderate COVID-19 in the period 03/2020–03/2022 participated in the study. Disease severity was evaluated according to the WHO criteria [22]. Patients were enrolled at the Department of Infectious Diseases of the Military Medical Academy and the Specialized Hospital for Active Treatment of Infectious and Parasitic Diseases, both in Sofia, Bulgaria. Whole blood samples were collected after the acute phase, longitudinally (2-time points) from 19 patients or at a single time point from 76 patients, 30–750 days post symptom onset (DPSO). A total of 107 samples were analyzed at the following average time points: 90 (30–135) DPSO, 27 samples; 180 (173–224) DPSO, 26 samples; 270 (238–330), 12 samples; 362 (331–375), 31 samples; 542 (400–750), 11 samples. During the follow-up period, only three donors (3.2%) had confirmed mild re-infection. The demographic and clinical characteristics of enrolled patients are presented in Table A1, Appendix A. In a limited number of donors tested <9 months and >9 months post symptom onset (PSO) (Table A2), a detailed flow cytometry analysis of virus-specific T cells was performed. This study was reviewed and approved by the institutional review board of the National Center of Infectious and Parasitic Diseases (NCIPD) and conducted according to the principles of the Declaration of Helsinki. Informed consent was obtained from all donors and patients.

Peripheral blood mononuclear cells (PBMCs) were isolated by density gradient centrifugation using Lymphocytes Separation Media (1.077 g/mL, Capricorn Scientific, Ebsdorfergrund, Germany). After isolation, the cells were either tested or cryopreserved in fetal bovine serum (FBS; Corning) with 10% dimethyl sulfoxide (DMSO; Sigma Aldrich, Saint Louise, MO, USA) until use.

### 2.2. Peptides

The following peptides were used for SARS-CoV-2 specific stimulation: peptide pools designed as overlapping peptides spanning sequences of the Spike (S1) or Nucleocapsid (N) proteins, including antigenic formulations of 253 peptides offering maximum epitope coverage for enhanced detection of T-cell reactivity and no HLA restrictions, both provided by Oxford, Immunotec.

The S1 protein of delta (B.1.617.2) variant, further denoted as “delta” is composed of 253 peptides, 15-mers overlapping by 10 amino acids, synthesized as crude material (TC Peptide Lab, San Diego, CA, USA), and individually resuspended in dimethyl sulfoxide (DMSO) at a concentration of 1 mg/mL. Megapools (MP) for each antigen were created by pooling aliquots of these individual peptides, as previously described [16].

### 2.3. ELISpot Assay (IFN-γ Enzyme-Linked Immunospot Assay)

ELISpot was used to detect T cells producing IFNγ after stimulation with virus-specific peptides (Immunotec, Oxford, UK). Freshly isolated PBMC were resuspended in an AIM-V medium containing Gentamicin Sulfate (10 µg/mL), L-Glutamine, and Streptomycin Sulfate (50 µg/mL) and seeded at 2.5 × 10^5^ cells/well in a 96-well plate, coated with anti-IFNγ in the presence 50 µL of peptide pools spanning sequence of the Spike (S1) or Nucleocapsid (N) proteins of SARS-CoV-2 (Immunotec, Oxford, UK).

AIM-V medium was used as a negative control for S1 and N-pools, an equimolar amount of DMSO (0.2%)-as a negative control for the delta pool, and PHA (phytohemagglutinin)-as a positive control. After 16 h incubation at 37 °C in a 5% CO_2_-humidified incubator, ELISpot was revealed following the manufacturer’s instructions. Briefly, PMBCs were washed four times with PBS, 200× concentered Conjugate Reagent (anti-IFN-γ mouse monoclonal antibody conjugated to alkaline phosphatase) was added to each well, and the plate was incubated at 2–8 °C for 1 h. After the incubation, the conjugate was discarded, and the wells were washed four times with PBS, followed by 7 min of incubation with Substrate solution (ready-to-use BCIP/NBT plus), covered at room temperature. The detection reaction was stopped using distilled water. Spot-forming cells (SFCs) were detected with an automated ELISpot reader (AID, Autoimmun Diagnostika, GmbH, Straßberg, Germany). The results were interpreted by the number of SFCs in the stimulated wells. After subtracting the number of SFCs in the negative control well, IFNγ+ T cells were calculated as the number of spots/2.5 × 10^5^ PMBCs per peptide-stimulated well and adjusted as the number of IFNγ-producing cells/10^6^ PBMCs. SFC ≥ 32/10^6^ PBMCs were considered as evidence of virus-specific response.

### 2.4. Activation-Induced Marker Assay 

PBMCs were isolated from heparinized peripheral blood following the protocol described before and seeded in a 96-well plate at a concentration of 2.5 × 10^5^ cells/well in RPMI-1640 medium, containing L-glutamine and sodium bicarbonate (Sigma-Aldrich, Saint Louise, MO, USA), and 10% FCS, stimulated with the three peptide pools S1, N (50 μL according to manufacturer’s instructions), and delta (2 μg/mL) for 18–20 h at 37 °C in a 5% CO_2_ atmosphere. RPMI-1640 medium containing 10% FCS was used as a negative control for S1 and N pools, and an equimolar amount of 0.2% DMSO-as a negative control for the delta pool. Both the negative controls and test samples were cultured in the presence of co-stimulator CD28/CD49d (cat# 347690, BD, Biosciences, San Jose, CA, USA), as already described [15]. At the end of the overnight incubation, cells were harvested according to stimulation conditions and washed four times with PBS.

### 2.5. Multi-Parameter Flow Cytometry

SARS-CoV-2 specific T-lymphocytes were identified by the expression of activation molecules using multi-parameter flow cytometry. The following directly conjugated monoclonal antibodies were used in the multi-color flow cytometry panel: anti-hCD3 BUV395 (clone SK7, cat# 564001, 1:40); anti-hCD4 BV786 (clone SK3, cat# 563877, 1:80); anti-hCD8 V-500C (clone SK1, cat# 647457, 1:80); anti-hCD45RA BUV496 (clone HI100, cat# 750258, 1:160); anti-hCD197 BB700 (clone 3D12, cat# 566437, 1:200); anti-hCD27 APC-R 700 (clone M-T271, cat# 565116, 1:40); anti-hCD95 BB515 (clone DX2, cat# 564596, 1:40); anti-hCD69 PE (clone L78, cat# 341652, 1:20); anti-hCD137 PE (clone 4B4-1, cat# 555956, 1:20); anti-hCD154 BV421 (clone TRAP1, cat# 563886, 1:40) (BD, Biosciences). LIVE/DEAD red fluorescent reactive dye Zombie Red Fixable dye (cat# 423110, 1:1000, BioLegend, San Diego, CA, USA), Table A3. Surface markers were detected by adding pre-titrated concentrations of directly conjugated antibodies resuspended in stain buffer (cat# 563794, BD, Biosciences), followed by 15 min incubation at room temperature, in the dark, and repeated washing with PBS. For each test, 1 × 10^6^ PMBCs were stained

Stained cells were acquired immediately on a FACSAria Fusion flow cytometer using FACSDiva software v.9.0.1 (BD Biosciences), and data were analyzed in FlowJo software v.10.8.1 (FlowJo, LLC, Ashland, OR, USA).

The gating strategy was as follows: singlet isolation (FSC-H versus FSC-A), live CD3 selection (CD3 versus LIVE/DEAD), lymphocyte enrichment (SSC-A versus FSC-A), CD4+ or CD8+ selection (CD4+ versus CD8+). CD3+/CD4+/CD8+ gating, with the identification of CD3+ CD4+ T cells and CD3+ CD8+ T cells. Activation-induced markers (AIM+) on CD4+ and CD8+ T-cells were determined according to the surface expression of CD137+, CD69+ and CD154+. To exclude false-positivity, the AIM+ populations were gated based on the negative controls: PMBCs cultivated in the presence of a co-stimulator and in the absence of peptides and stained in the same way as test samples. AIM+ CD4+ and CD8+ T-cells were further analyzed according to the expression of CD45RA and CD197 as central memory (CM, CD45RA- CD197+), naïve (CD45RA+ CD197+), effector memory (EM, CD45RA-CD197-), and terminal effector (TE, CD45RA+ CD197-) subsets. Total T_SCM_ was determined as a part of the subpopulation of T cells with naïve phenotype co-expressing CD95+ and CD27+ receptors (CD45RA+CD197+CD95+CD27+). Virus-specific T_SCM_ was defined as a part of the CD45RA+CD197+CD95+CD27+ T_SCM_ population expressing CD137 and/or CD69 and/or CD154 activation markers after SARS-CoV-2 specific stimulation. Detailed gating strategies for AIM+T and T_SCM_ are depicted in the relevant figures.

### 2.6. Statistical Analysis 

Comparisons between groups were performed with a one-tailed unpaired Student t-test. For data sets that did not pass Shapiro-Wilk or D’Agostino-Pearson normality tests, Mann–Whitney U test for two unpaired groups (MW) test was used. Data are, respectively, presented as means (±standard deviation, STD) or median (min, max). For both parametric and non-parametric tests, post-test comparisons were used to compare specific groups. *p*-values less than 0.05, at CI 0.95, were considered significant. Analyses were performed using GraphPad Prism v.9.

## 3. Results

### 3.1. SARS-CoV-2-Specific IFNγ + T Cells Significantly Decrease beyond 9 Months after the Infection

**Figure 1:**
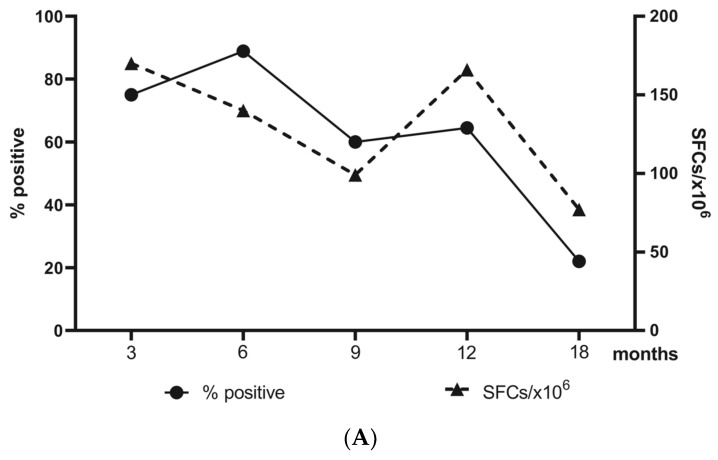

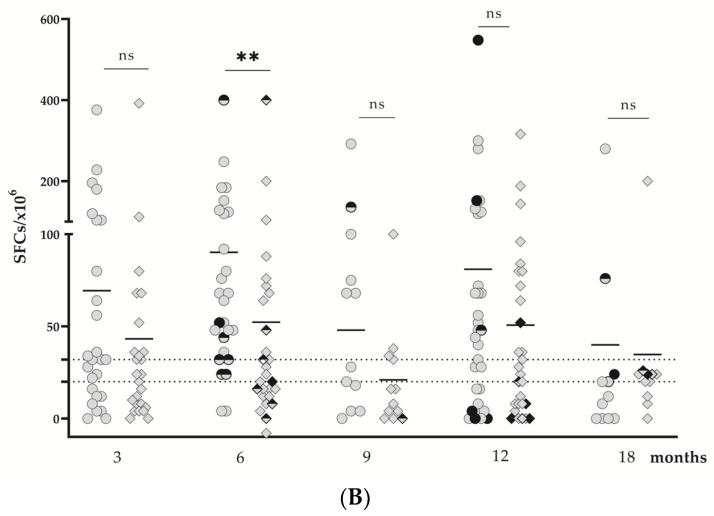
Dynamics of SARS-CoV-2-specific T-cell responses until 18 months post-infection. (**A**) Share of positive responses (thick line) and mean numbers of SFC/10^6^ PMBCs (dotted line). (**B**) Individual values for the number of S1- (circles) and N- (diamonds) specific IFN-γ secreting T cells. The dotted line corresponds to the cut-off level (32 SFCs/10^6^ PMBCs). Donors vaccinated within 6 months before sampling are designated with full black circles, and those vaccinated beyond 6 months before sampling with semi-black circles, with either BNT162b2 (Pfizer-BioNTech) or ChAdOx1 (Oxford/AstraZeneca); ** *p* < 0.01.

To evaluate the dynamics of virus-specific T-cell response, we first applied ELISpot IFNγ assay after overnight stimulation of PBMCs obtained at different time points after convalescence, as described in Materials and Methods. Stimulation was performed with either S1 or N-peptide pools derived from the ancestral variant. The cumulative share of positive responses (to S1 and/or N) reached a maximum of 89% six months after the infection, followed by a gradual decrease so that at 18 months, only 22% of tested samples responded with a virus-specific expression of IFNγ (Figure 1A).

The same tendency was observed for the average number of SFCs (Figure 1A) which decreased practically twofold, from 170/10^6^ PBMCs at 3 months to 77/10^6^ PBMCs at 18 months. Unlike the share of responding samples, the average number of detected SFCs increased beyond 9 months post-infection. To analyze the relative implication of S1- and N-specific responses for the observed particular curve, we compared the average number of SFCs in response to S1 and in response to N before and beyond 9 months post-infection (Supplementary Figure S1). No significant difference was detected between the samples acquired before and beyond 9 months post-infection, neither for S1- nor for N-specific responses. However, S1-specific responses were significantly stronger as compared to N-specific ones, and this difference was observed only before 9 months post-infection. To check the possible effect of vaccinations, we analyzed in more detail the response to S1 and N peptide pools (Figure 1B). The epitope-specific analysis showed that the decrease of SARS-CoV-specific T-cell responses at 9 months was entirely due to the quickly decreasing reactivity to the N-peptide pool. At the same time, S1-specific T-cell responses remained quasi-unchanged 12 months post-infection (mean SFCs/10^6^ PBMCs at the studied time points: 70; 90; 68; 80; and 40). In fact, at all studied time points, the average number of N-specific T- cells was lower as compared to the S1-stimulated response; this difference was already significant at 6 months (52 vs. 90, MW *p* < 0.01). A total of 16 donors reported vaccination at different time intervals after COVID-19 (Figure 1B). The analysis of S1-specific responses of donors that had been additionally vaccinated (Supplementary Figure S2) did not show significant differences as compared to the rest of the donors. Additionally, the time interval since vaccination did not seem to have a significant effect on the strength of SARS-CoV-2 specific responses at 6 and 12 months. In fact, 3/7 recently vaccinated and 5/9 vaccinated more than 6 months ago donors had positive T-spot results (Figure 1B). We concluded that the observed increase at 12 months post-infection was rather due to subsequent asymptomatic infections than to vaccination. Overall, these results indicated that the number of circulating IFNγ+ virus-specific T cells is not an adequate marker of long-term memory for SARS-CoV-2, especially in non-epidemic settings.

### 3.2. SARS-CoV-2-Specific Memory T Cells Are Detectable beyond 9 Months after the Infection

**Figure 2:**
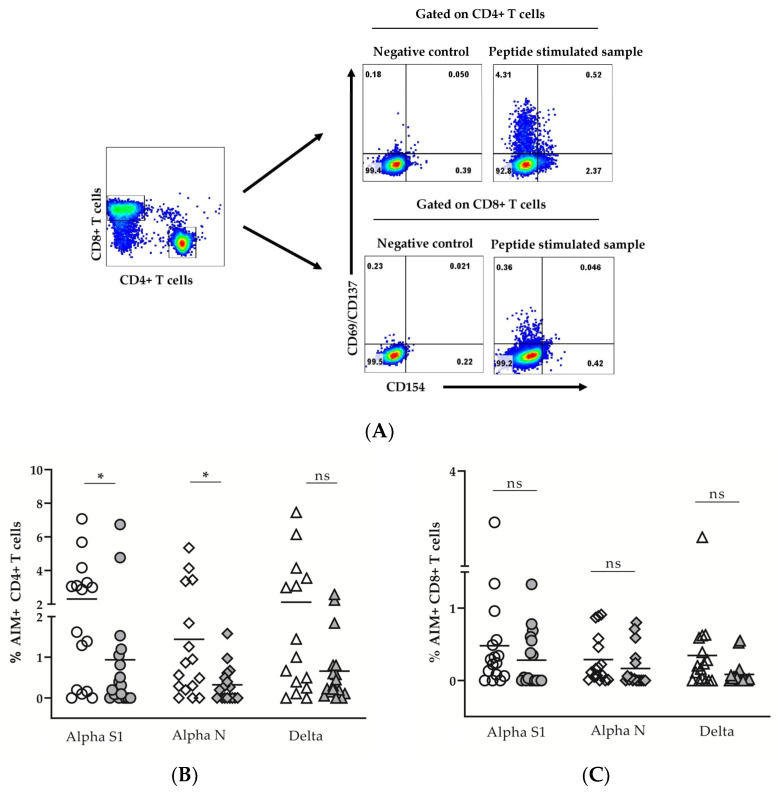
Flow-cytometry detection of AIM+CD4+ and AIM+CD8+ T cells. Gating strategy (**A**). The percentage of AIM+ (CD69+CD137+ and/or CD154+) virus-specific cells within gated CD4+ and CD8+ T cells was determined after subtracting the values obtained for the negative control. Individual values for AIM+CD4+ (**B**) and AIM+CD8+ T cells (**C**), specific to peptide pools from alpha S1 (circles), alpha N (diamonds) and delta (triangles) variants. The responses before (open symbols) and beyond (grey symbols) 9 months after the infection are compared. (* *p* < 0.05; ns, non-significant; MW).

The quick IFNγ expression is inherent only to the effector and a part of the effector-memory T cells. To characterize more thoroughly the memory virus-specific T-cell response in a limited number of convalescent donors (*n* = 33, Table A2), we performed flow cytometry AIM analysis of in vitro stimulated T cells. Representative density plots about the gating strategy and individual values for AIM+CD4+ and AIM+CD8+ T cells are given in Figure 2A–C.

**Figure 3:**
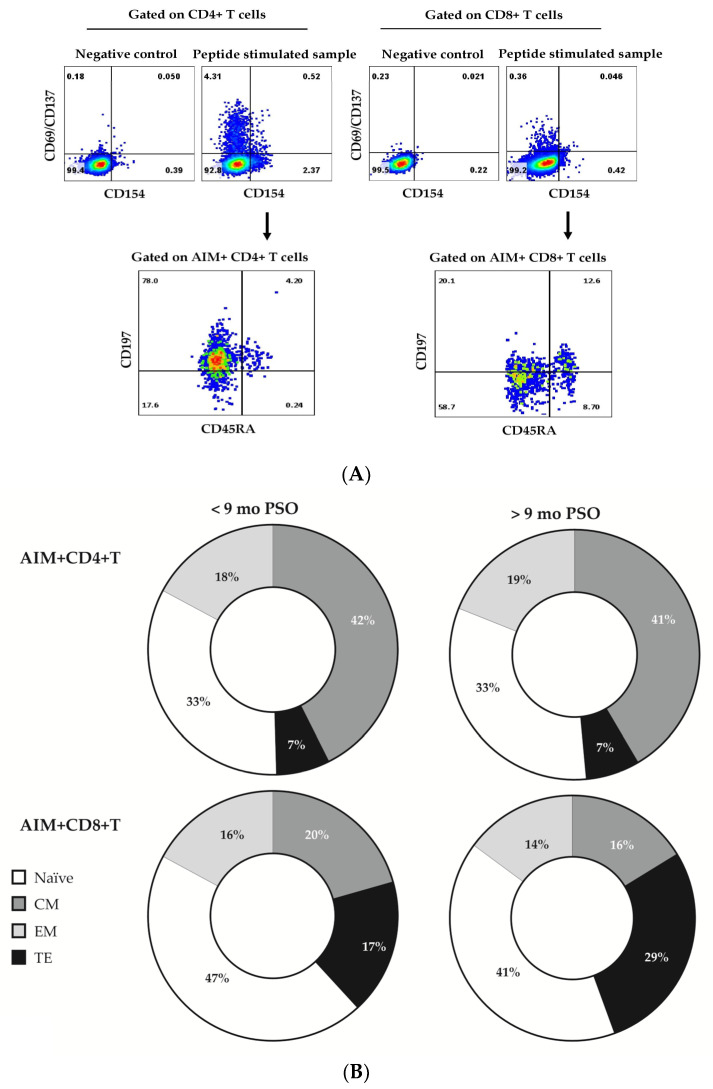

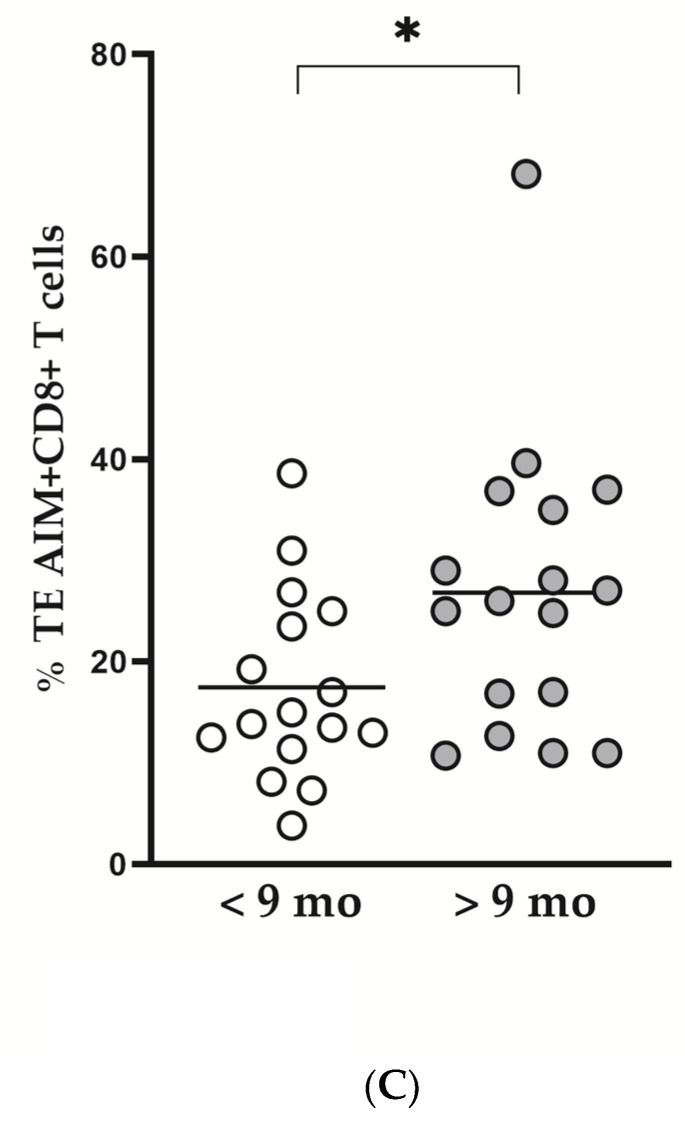
Comparison between the differentiation profiles (relative shares of naïve, CM, EM, and TE) among the S1–specific T cell pools. (**A**) Gating strategy After detection of AIM+ (CD69+CD137+ and/or CD154+) CD4+ and CD8+T cells, as explained in Figure 2, the shares of naïve (CD45RA+CD197+), CM(CD45RA-CD197+), EM(CD45RA-CD197-), TE(CD45RA+CD197-) were determined within gated AIM+CD4+ or AIM+CD8+ T cells. (**B**) Relative shares (mean values) of naïve, CM, EM, and TE cells among AIM+CD4+ (upper panel) and AIM+CD8+ (lower panel) S1-specific T-cell pool in samples < 9 mo (left side) and > 9 mo PSO (right side). (**C**) Individual values for the share of TE cells among AIM+CD8+ T in samples <9 mo and >9 mo PSO. Statistical differences were evaluated by non-paired *t*-test.

We compared the responses of two groups of donors: less than 9 months (average 171 days) and beyond 9 months (average 454 days) after the infection, infected during two periods corresponding to the prevalence of the ancestral/alpha or of delta variant in Bulgaria (Table A2). The stimulation was performed with either S1- or N-peptide pools from the original SARS-CoV-2 or S1 from the delta (B 1.617.2) variant. A significant decrease of S1- and N-specific CD4+ T cells was observed beyond 9 months (MW *p* < 0.05, and *p* < 0,05, respectively, Figure 2B). The same tendency was observed for delta-specific AIM+ CD4+ T though it did not reach statistical significance (MW *p* = 0.06), Figure 2B. Importantly, no significant decrease of AIM+CD8+ T cells was observed beyond 9 months post-infection as compared to the earlier period (MW *p* > 0.05, Figure 2C). The frequency of AIM+CD8+ T, regardless of their specificity and the period tested (Figure 2B,C), was lower as compared to AIM+CD4+. The mean % of AIM+CD8+ varied between 0.12% and 0.48% from the CD8+ pool, while AIM+CD4+ were between 0.3% and 2.5% from the CD4+ pool. Only one donor tested more than 9 months post-infection was sampled before 08/2021 (i.e. before the Delta variant was detected for the first time in Bulgaria). Nevertheless, he responded to the delta pool (0.15% AIM+CD4+ T cells and 0.59% AIM+CD8+ T cells), which could be expected based on the similarity of the variants. To better explain these results, we analyzed the differentiation profiles of virus-specific (AIM+) CD4+ and CD8+ T cells according to the co-expression of CD45RA and CD197 receptors (Figure 3A–C).

The profiles of S1-, N-, and -specific T were not significantly different. Therefore, only S1-specific cells are shown. While AIM+ CD4+ T cells were mostly of CM phenotype (average 42% and 41% for groups <9 mo and >9 mo post-infection, respectively), AIM+ CD8+ T cells were mostly naïve-like (average 47% and 41% for groups <9 mo and >9 mo post-infection, respectively). The comparison of the differentiation profiles before and after 9 months post-infection did not show significant differences for most of the subsets. TE AIM+CD8+ was the only subset that increased significantly after 9 months post-infection (average 29% vs. 17%, *p* < 0.05), indicating the importance of CD8+ T-cell effector mechanisms for protection in case of subsequent challenges by SARS-CoV-2 antigens (Figure 3C). 

**Figure 4:**
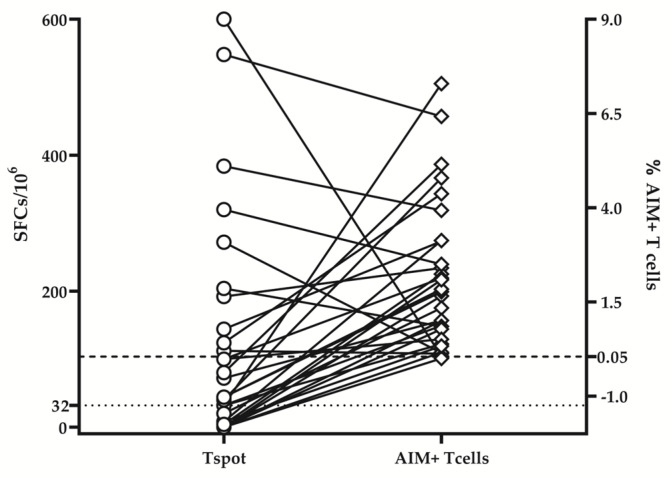
ELISpot and flow cytometry results regarding memory T-cell responses. Comparison between the share of virus-specific memory T cells determined by ELISpot and by flow cytometry detection of AIM+ T cells (paired data). Both samples acquired before and beyond 9 months post-infection are included. The dotted and dashed lines correspond to the cut-off levels of ELISpot (32 SFCs/10^6^ PMBCs) and flow cytometry AIM test, respectively.

At the same time, the relatively small share of E/TE CD4+ and CD8+ virus-specific T corroborated with the low share of positive results in IFNγ ELISpot assay beyond 9 months post-infection. As shown in Figure 4, the share of positive T-cell responses detected by IFNγ -ELISpot was considerably lower than the share of positive responses of the same donors in AIM+ flow cytometry assay: 63% (19/31) vs. 97% (30/31).

Therefore, flow cytometry detection of AIM+ T cells seems a more adapted method for the detection of virus-specific immune memory in the long term.

### 3.3. Circulating SARS-CoV-2 Specific CD8+ T_SCM_ Are a Reliable Biomarker of Long-Term Protection 

**Figure 5:**
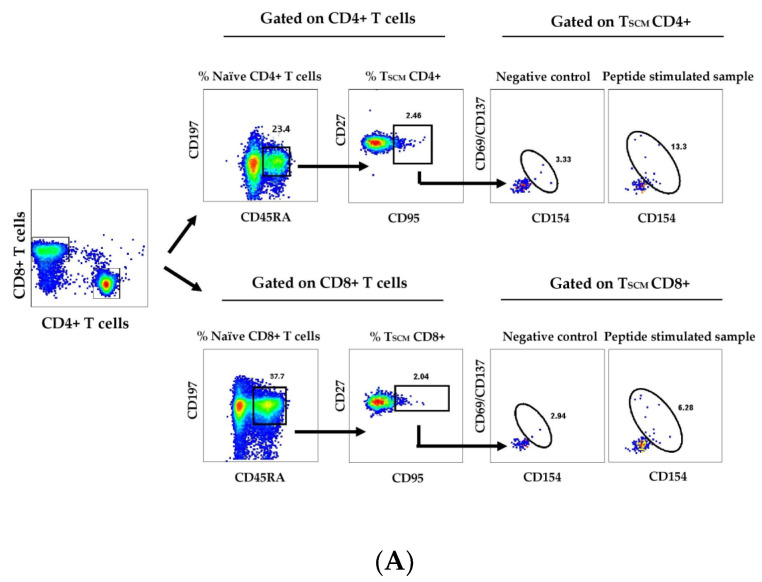

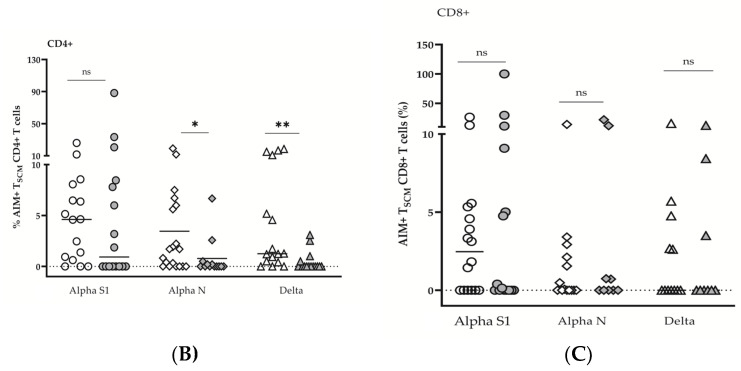
Detection of SARS-CoV2-specific CD4+ and CD8+ T_SCM_ Gating strategies (**A**). CD4+ and CD8+ T_SCM_ were determined as the share of naïve (CD45RA+CD197+) CD4+ or CD8+ T cells, co-expressing CD27 and CD95. Frequency of SARS-CoV-2 specific T_SCM_ among CD4+ T_SCM_ (**B**) and among CD8+ T_SCM_ cells (**C**) The frequency of T_SCM_ (CD45RA+CD197+CD27+CD95+) was determined after stimulation with the relevant peptide pools as described in Figure 2 as AIM+(CD137+CD69+CD154+) cells. Designations: T_SCM_ specific to alpha S1 (circles), alpha N (diamonds) and delta (triangles) peptides, up to 9 months (open symbols) and beyond 9 months (grey symbols) after the infection. Statistical significance is determined by the Mann–Whitney test (ns, not significant, *p* > 0.05; * *p* < 0.05; ** *p* ≤ 0.0001).

The important share of AIM+CD4+ and CD8+ T cells with the naïve-like phenotype (CD45RA+CD197+) prompted us to study the circulating pool of T_SCM_ cells. The total pool of T_SCM_ was evaluated as the percentage of CD4+ and CD8+ T cells (the gating strategy is shown in Figure 5A) that was comparable before and beyond 9 months post-infection. (mean 0.5% and 0.8%; 0.43% and 0.7% of CD4+ and CD8+ T-cell pools, respectively). Virus-specific CD4+ and CD8+ T_SCM_ were further analyzed as a proportion of AIM+ cells from the respective T_SCM_ pool. According to our results, AIM+CD4+ T specific for alpha S1 peptides did not decrease significantly beyond 9 mo post-infection, unlike alpha N-specific and delta-specific CD4+ T_SCM_ (Figure 5B).

In separate donors, we detected importantly increased S1-specific CD4+ T_SCM_ at the later stages post-infection and those who were not previously vaccinated. Importantly, both alpha- and delta-specific CD8+ T_SCM_ pools were maintained in the long term, also with substantial increases in individual frequencies (Figure 5C). Again, those who were not vaccinated donors.

Interestingly, the single donor tested > 9 mo PSO who was sampled before the appearance of the Delta variant in Bulgaria did not display delta-specific T_SCM_ while having delta-specific CD4+ and CD8+ T cells.

**Figure 6:**
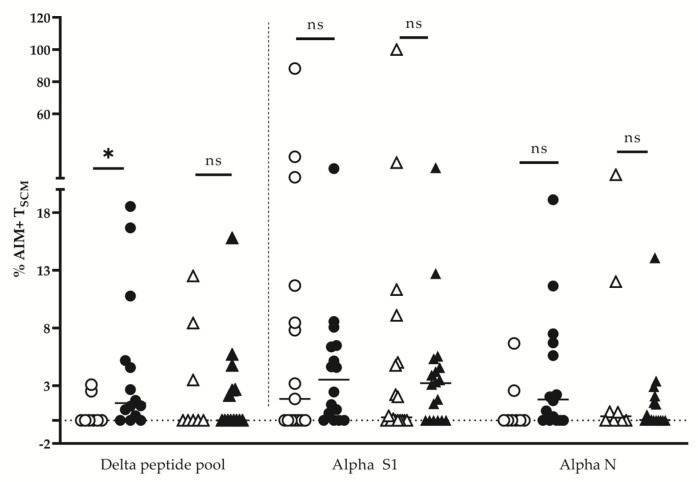
Effect of the priming variant on the proportions of T_SCM_. The proportions of CD4+ (circles) and CD8+ (triangles) SARS-CoV-2 specific T_SCM_ among all T_SCM_ (CD45RA+CD197+CD27+CD95+) in donors primed with alpha variant (open symbols) and in donors primed with delta variant (black symbols) were compared after stimulation with Delta peptides (left part) or S1 and N peptides (right part). Statistical significance was determined by the Mann-Whitney test (* *p* < 0.05; ns, not significant).

A possible bias of this analysis could follow from the fact that in the group <9 mo PSO included mostly donors infected with delta-variant, while those in the group >9 mo PSO were infected at the beginning of the pandemic with alpha SARS-CoV-2. To exclude a possible effect of the priming antigen on the longevity of T-cell memory, we compared the proportions of alpha- and delta-specific CD4+ and CD8+ T_SCM_ (CD45RA+ CD197+CD27+CD95+) in donors primarily infected with alpha vs. those primarily infected with delta (Figure 6).

**Figure 7:**
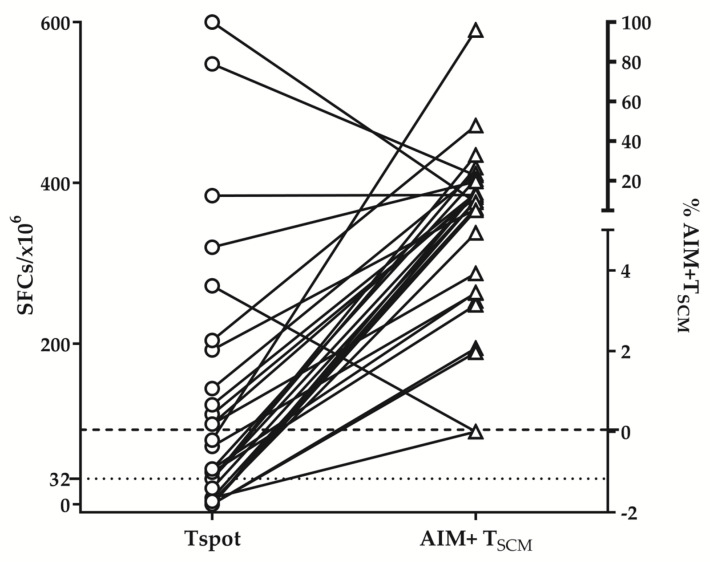
ELISpot and flow cytometry results regarding memory T-cell responses. Comparison between the share of virus-specific memory T cells determined by ELISpot and by flow cytometry detection of AIM+ T_SCM_, paired data. Both samples acquired before and beyond 9 months post-infection are included. The dotted and dashed lines correspond to the cut-off levels of ELISpot (32 SFCs/10^6^ PMBCs) and flow cytometry AIM test, respectively.

While the proportion of CD4+ T_SCM_ responding to delta-peptide stimulation was significantly higher in donors primed with the delta variant, no significant differences were found for CD8+ T_SCM_ pools. Thus, circulating SARS-CoV-2 specific T _SCM_ cells were a better biomarker of long-term protection and outweighed IFNγ ELISpot in a paired comparison (Figure 7).

Our results also show that both CD4+ and CD8+ virus-specific T_SCM_ (CD45RA+CD197+CD27+CD95+) pools are stably maintained and may proliferate and back-differentiate into effectors when needed. Finally, we propose that in the settings of circulating virus, CD8+ T_SCM_ are primarily engaged in delivering immediate protection.

## 4. Discussion

Three years after the beginning of the pandemic, the duration and qualities of immune protection in the settings of constantly evolving pathogens remain an open question. Using IFNγ ELISpot and flow cytometry, we compared virus-specific CD4+ and CD8+ T-cell responses over 18 months post-infection with ancestral, alpha or delta SARS-CoV-2 variants and demonstrated that CD8+ T_SCM_ is a relevant marker of long-term protection.

Antigen-primed naïve CD4+ and CD8+ T cells are activated and differentiate into effectors that eliminate infected target cells, employing largely IFNγ-mediated cytotoxicity [23]. Hence, IFNγ-release assays (IGRAs) were widely employed to assess SARS-CoV-2 specific T-cell response. A number of studies demonstrated robust T-cell responses detectable early in COVID-19 patients (one week after exposition) and associated with successful recovery in convalescent donors [10,24,25]. Overall, circulating SARS-CoV-2-specific CD8+ T cells were less consistently observed than CD4+ T cells [12,24,26]. We and others have demonstrated that IFNγ+ virus-specific T cells are stable for 3 months and detectable at least 6 months after natural exposure or immunization [7,27]. In a study of 188 convalescent donors up to 178 DPSO, SARS-CoV-2-specific CD4+ and CD8+T-cells’ half-life was extrapolated to be 3 to 5 months [14].

In the present study, almost three years after the beginning of the pandemic, we had the possibility to look at SARS-CoV-2-specific T cell responses over 540 DPSO and demonstrated well detectable IFNγ+ responses (in 65% of donors) one-year post-infection, with sharp decline at 18 months PSO. Importantly, we documented significant differences associated with epitope specificity of virus-specific T cells: S1-specific responses were clearly dominant in line with earlier studies [26] and were stable for at least one year, while N-specific IFNγ+ T cells practically disappeared already at 9 months PSO. At the same time, we showed that restimulation due to contact with continuously circulating virus strains can maintain an active pool of both S1- and N-specific T cells beyond 9 months post-infection. Moreover, no significant difference in the strength of S1 and N-specific responses was observed at the later time points, indicating that N-specific responses were possibly less affected by the ongoing mutations.

Immune memory is an obligatory component of protective immunity mediated by long-living antigen-specific cells, generally assigned to the broad central memory (CM) subset by virtue of their post-activation (CD45RA−) state, recirculation ability (CD197+) and high proliferation index at the expense of function. CM cells differentiate into effectors (E), passing through several transitional stages (TM1, TM2, EM) with increasing functional capacity [19]. Therefore, it was not surprising that IFNγ+ cells tend to decrease significantly with time PSO.

It is well known that the phenotype of circulating pathogen-specific T cells largely varies, depending on multiple factors but mostly the characteristics of the pathogen, the initial antigen load and its clearance. In our hands, the presence of virus-specific cells with E and EM phenotype at the later stages of the study was rather due to subsequent asymptomatic reinfections and only in single cases to vaccination or symptomatic reinfection. The significant increase of S1-specific responses observed at 12 months coincided for most of the donors with the period Oct 2021–Jan 2022, when an important rise of COVID-19 cases was observed in Bulgaria. On the other hand, in settings with low virus circulation, one might expect the dominance of CM cells, as observed by Dan et al. [14]. Even in our hands, the virus-specific T cells with E and EM phenotypes were a minor part of the SARS-CoV-2-specific pool.

Therefore, we and others have discussed the necessity of applying modified protocols adapted to detect “genuine” memory cells. Thus we proposed that extended activation (96 h instead of overnight increases significantly the share of positive response [27], while Grifoni et al. [26] and Dan et al. [14] made use of AIM expression in response to SARS-CoV-2 peptide mega pools in vitro. Tarke et al., employing AIM detection after in vitro stimulation, proved that 85% of CD8+ and 80% of CD4+ T-cell responses to the original and to subsequent variants were preserved 6 months after the induction (vaccination) [16]. In the present study, by utilizing AIM detection after in vitro stimulation, we revealed consistent virus-specific CD4+ and CD8+ T-cell responses over 9 months post-infection in 97% of tested donors as compared to 63% in ELISpot. In addition, the AIM assay permits the separate analysis of CD4+ and CD8+ responses. In our hands, unlike CD4+, CD8+ AIM+T cell virus-specific responses did not decline in the long term, implying faster and more operational protection provided by this part of cellular immunity. This protective effect was supported by a very low incidence of reinfections (3.2%) and the absence of severe COVID-19 cases during the follow-up. Even more, terminally differentiated (TE) CD8+ AIM+ T significantly increased after 9 months PSO (Figure 3B).

After antigen removal, SARS-CoV-2-reactive T cells can acquire a variety of memory phenotypes in lymphoid and peripheral tissues. According to our results, the differentiation profiles of CD4+ and CD8+ virus-specific T cells were not identical, with CD4+ T cells being largely in the CM pool, while CD8+ T cells were naïve-like, partially corroborating with previous studies [10,15,19].

The majority of current data suggests that there is a CD4+ T cell bias towards CM and a CD8+ T cell bias towards TEM, with heterogeneity in the differentiation status. However, most published data reported on the differentiation profiles of SARS-CoV-2-specific T cells during the acute phase of infection or in recently recovered patients up to 2–3 months PSO [12,25,28]. Peng described a prevalent CM phenotype for CD4+ SARS-CoV-specific cells, whereas CD8+ memory T cells were early differentiated memory (CD197+ CD127+ CD45RA−/+) or TEMRA with preserved proliferative potential (CD127+) [10,12,26,29,30].

To our knowledge, the present study extends for the first time these observations beyond 9 months PSO. Earlier studies have mostly looked for an association between the phenotype of virus-specific T cells and disease severity and/or epitope specificity. In studies of mild to severe COVID-19, CD8+ TEM/TEMRA cells appeared to be less differentiated compared to critical cases, including those with acute respiratory distress syndrome (ARDS). A higher proportion of CD8+ T-cell responses was observed in mild disease, suggesting a potential protective role of CD8+ or a pathogenic role of CD4+ T-cell responses in severe disease [11,19].

In our hands, the memory T cell specificity (S1, N, or Delta) was not associated with a specific differentiation subset. We looked instead for a possible evolution of the differentiation status of the virus-specific pool. The only significant difference beyond 9 months post-infection was a relative increase in the TE CD8+ pool. Since all donors were asymptomatic at that time, this evolution could be a token of antigenic challenge in the settings of intensive viral circulation. This also implies the persistence in the virus-specific pool of long-living and ready-to-differentiate progenitors as the stem-like memory cells.

The idea of a stem-cell-like memory subset subtype, which can self-renew and generate central memory and effector memory T cells when needed, developed only during the last decade [20,31]. In the context of the COVID-19 pandemic, T_SCM_ was evoked by a few studies. Kared et al. reported that SARS-CoV-2–specific CD8+ T cells displayed unique phenotype, enriched in SCM and TM2 cells as a possible key to durable protection. They looked at convalescent donors, a median of 42.5 days from initial diagnosis [19]. Jung et al. evaluated SARS-CoV-2-specific CD4+ and CD8+ T-cell responses over 10 months post-infection, detecting an increased proportion of T_SCM_ cells between 60 and 120 DPSO [15]. Our study further extends these conclusions confirming that stem-cell-like memory to SARS-CoV-2 is sustained and operational beyond 18 months PSO, as witnessed by the increased individual values of AIM+ CD8+ and the significantly increasing share of CD8+ T with TE phenotype. At the same time, the proportions and specificities of individual Tscm pools may vary a lot depending on the context of epidemiological settings and contacts. 

An extremely important aspect of T-cell memory to SARS-CoV-2 is the relative contribution of T-cell clones with different antigen specificity to the overall protective effect. This issue is related both to the concept of herd immunity and to vaccine construction. In the settings of constantly evolving pathogens and emerging variants, it is primordial to evaluate the fitness of existing T_SCM_ clones to the new antigenic challenges.

Previous studies have mostly compared CD4+ and CD8+ T-cell responses to S1, N, M, ORF3a and nsp antigens of the ancestral virus. In fact, T-cell responses have been detected against most SARS-CoV-2 proteins (21/24), proportional to their expression level [26]. Jung et al. compared in detail the longevity and phenotype evolution of T-cell pools specific for S, N and M antigens both in IFNγ. ELISpot and AIM detection assays and concluded that those were stable and without significant differences during the convalescent phase [15]. Unlike them, we observed a much more quickly declining N-specific as compared to S1-specific IFNγ response and a considerably lower number of N-specific SFCs (Figure 1B). On the other hand, S1- and N-specific responses evaluated by AIM+ CD4+ and CD8+ T were quite similar, in line with Jung’s data [15]. Once again, these results corroborate the fact that IGRA-generated information depends very much on recent antigen stimulations and hence on the particular epidemiological settings of the study. In Bulgaria, a number of factors as the low vaccination rates and non-compliance to physical measures, brought a continuous and intensive circulation of the virus, unlike the situation in South Korea [15].

Soon after the worldwide spread of the original SARS-CoV-2, viral variants began to emerge, characterized by mutations affecting mostly the S gene19, encoding for the Spike protein [32]. The Alpha variant (B.1.1.7) was the first labeled variant of concern (VOC) with 29% increased transmissibility and signs of immune escape [33]. Followed the Delta variant (B.1.617.2), which rapidly became the dominant strain in the world, thanks to 97% increased transmissibility and significant immune escape effect [34,35,36]. In Bulgaria, alpha VOC was detected in June 2020 and dominated during the period of the second and the third wave until 2021, when Delta was detected for the first time [37] and was the leading cause of morbidity and mortality during the fourth and fifth wave. Therefore, it was important to test whether T-cell memory generated during the dominance of alpha VOC was operative against Delta and to what extent the routine methods for assessment of T-cell memory were affected.

Following the genetic evolution of SARS-CoV-2, several large studies tested the escape effect of the new mutations on the efficiency of previously generated humoral and T-cell responses. Overall, while significantly affecting antibody-mediated immunity, T-cell responses seemed to a great extent intact [38]. A large study by Tarke et al. assessed the impact of alpha, beta, gamma, delta and omicron mutations on T-cell memory generated by vaccines based on the ancestral virus and documented 85 to 100% preservation of responses [16]. In our hands, the proportions and particular specificities of T_SCM_ pools obviously depended on the individual epidemiological context of their formation. However, in line with the above studies, both alpha- and delta-specific CD8+ responses generated were viable more than 9 months after the initial infection, thanks to a circulating subset of CD8+ TSCM, and did not seem to depend on the priming variant.

A major limitation of the study is the small number of samples analyzed for the presence of SARS-CoV-2-specific T_SCM_ cells. Their relevance as a marker of long-term protection should be validated in further large-scale prospective studies, including analysis of their possible heterogeneity and functionality.

## 5. Conclusions

Our results demonstrate that: beyond 9 months post-infection, all tested patients displayed AIM+ virus-specific T cells unlike IFN-γ + virus-specific T cells; SARS-CoV-2 specific AIM+T cells were mostly of naive-like phenotype; The proportion of AIM+ CD8+T _SCM_ was stable and responded similarly to stimulation with peptides from the priming variant, as from the later alpha or delta variants.

Based on these observations, beyond 9 months, post-infection virus-specific AIM+ CD8+ T_SCM_ cells may indicate long-term protection from COVID-19. Therefore, they are sensitive, lasting, and so far independent from the viral evolution indicator of protection against SARS-CoV-2 infection.

## Figures and Tables

**Figure A1:**
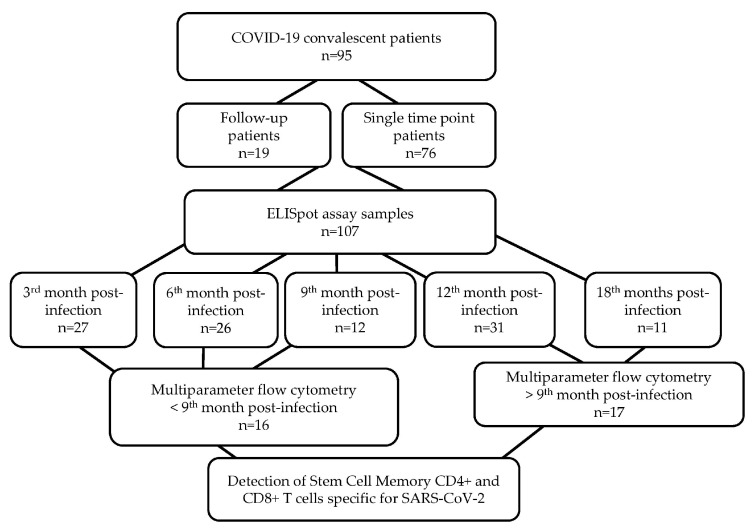
Flow chart of participants and experiments performed.

## Data Availability

Requests for access to the study data can be submitted to the corresponding author.

## Supplementary Materials

The following supporting information can be downloaded at: https://www.mdpi.com/article/10.3390/diagnostics13071280/s1, Figure S1: Individual values for the number of S1- (circles) and N- (triangles) specific IFN-γ secreting T-cells. The dotted line corresponds to the cut-off level (32 SFCs/10^6^ PMBCs). The responses <9 mo and >9 mo PSO are compared.; Figure S2: Individual values for the number of S1- specific IFN-γ secreting T-cells in non-vaccinated and vaccinated donors. The dotted line corresponds to the cut-off level (32 SFCs/10^6^ PMBCs). The responses at 6 mo and 12 mo PSO are compared.

## Appendix A

**Table A1:** Characteristics of 95 donors included in the study.

DPSO (Med; Min-Max)	90 30–135	180 173–224	270 238–330	362 331–375	542 400–750
Samples (n)	27	26	12	31	11
Age [years, med; min-max]	59 19–68	53 24–71	47 29–63	56 32–69	51 35–63
Sex (male/female)	12/15	10/16	2/10	15/16	6/5
Vaccinated after infection (n)	0	7	1	6	2
Period of infection m/yy	3/20–1/22	7/20–2/22	6/20–10/21	6/20–3/21	9/20–3/21
% positive IFNγ ELISpot results	75	89	60	65	22

**Table A2:** Characteristics of groups tested less than 9 months and beyond 9 months after the infection.

	<9 mo PSO	>9 mo PSO
Participants [n]	16	17
Age [years, med; min-max]	53 (27–66)	53 (35–69)
Sex [M/F]	5/11	7/10
Vaccinated after infection (n)	12	4
DPSO (med; min-max)	171 (30–270)	454 (330–750)
Period of infection m/yy	September 2021 March 2022	March 2020 March 2021
Dominant variant at the time of infection	delta variant	ancestral/alpha variant

**Table A3:** The following monoclonal antibodies were used for multi-color flow cytometry.

Monoclonal Antibodies	Fluorochrome	Source	Identifier
anti-hCD3, clone SK7	BUV395	BD, Biosciences	cat# 564001
anti-hCD4, clone SK3	BV786	BD, Biosciences	cat# 563877
anti-hCD8, clone SK1	V-500C	BD, Biosciences	cat# 647457
anti-hCD45RA, clone HI100	BUV496	BD, Biosciences	cat# 750258
anti-hCD197, clone 3D12	BB700	BD, Biosciences	cat# 566437
anti-hCD27, clone M-T271	APC-R700	BD, Biosciences	cat# 565116
anti-hCD95, clone DX2	BB515	BD, Biosciences	cat# 564596
anti-hCD69, clone L78	PE	BD, Biosciences	cat# 341652
anti-hCD137, clone 4B4-1	PE	BD, Biosciences	cat# 555956
anti-hCD154, clone TRAP1	BV421	BD, Biosciences	cat# 563886
anti-hCD28/CD49d, clone L293/clone L25		BD, Biosciences	cat# 347690
