# Supplementary material for: Virus-Specific Stem Cell Memory CD8+ T Cells May Indicate a Long-Term Protection against Evolving SARS-CoV-2
Source: Diagnostics (Basel). 2023 Mar 28;13(7):1280. doi: 10.3390/diagnostics13071280 (PMC10093371; doi:10.3390/diagnostics13071280)
Supplement: Supplementary file 1 [file diagnostics-13-01280-s001.zip › diagnostics-2134014-supplementary.pdf]

Supplementary data

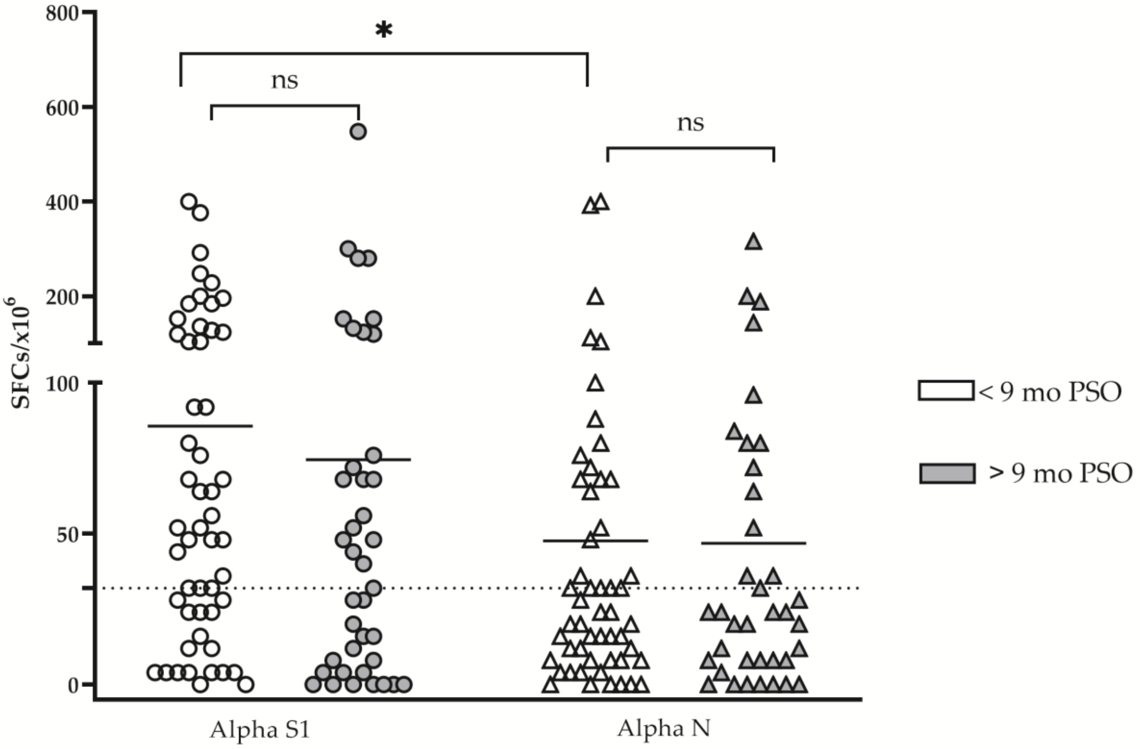

Figure S1. Individual values for the number of S1- (circles) and N- (triangles) specific IFN-γ secreting T-cells. The dotted line corresponds to the cut-off level (32 SFCs/106 PMBCs). The responses <9 mo and >9 mo PSO are compared.

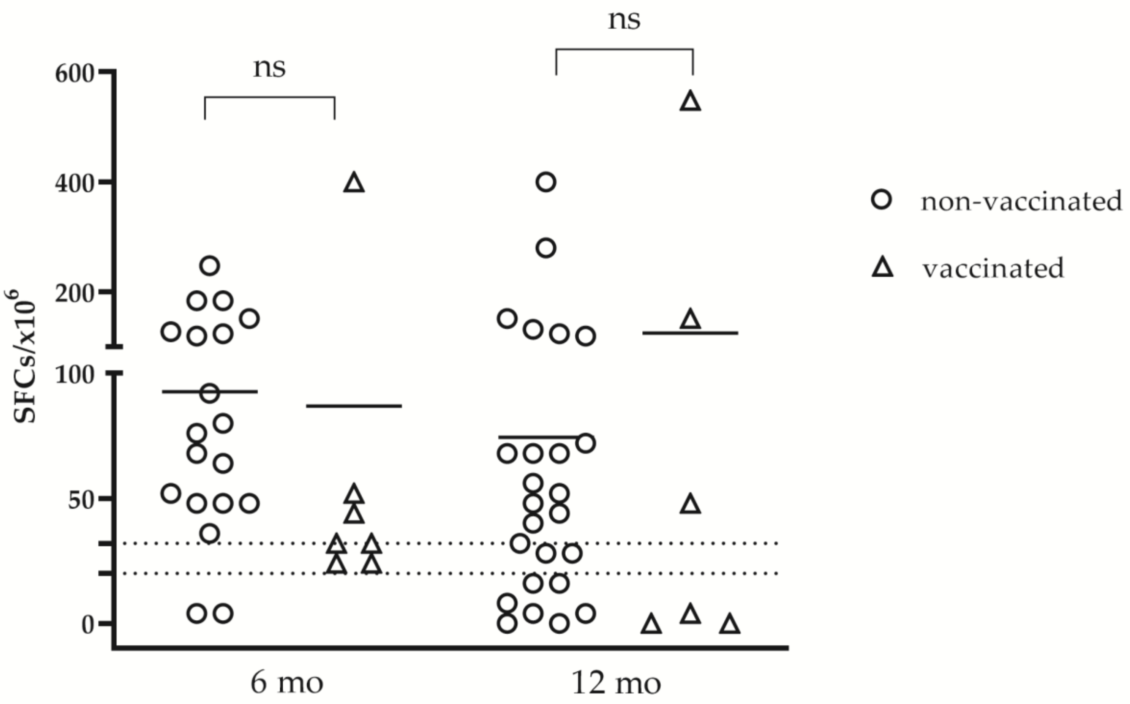

Figure S2. Individual values for the number of S1- specific IFN-γ secreting T-cells in non-vaccinated and vaccinated donors. The dotted line corresponds to the cut-off level (32 SFCs/10 6PMBCs). The responses at 6 mo and 12 mo PSO are compared.
